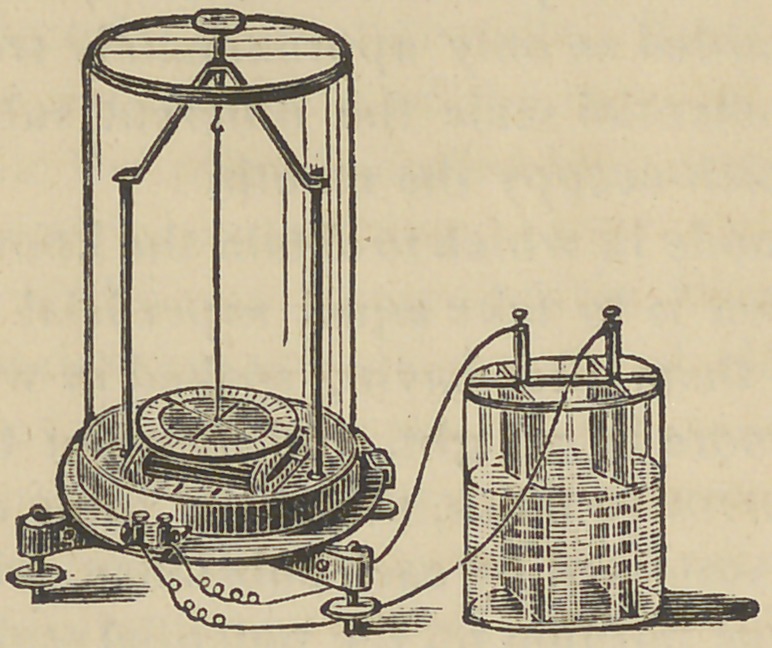# Oral Electricity

**Published:** 1876-07

**Authors:** Henry S. Chase

**Affiliations:** St. Louis


### ORAL ELECTRICITY.

BY HENRY S. CHASE, M. D., ST. LOUIS.

*Read before the Illinois, Iowa and Missouri State Dental Societies.*

The oral cavity may be compared, in some sense, to a single cell of a voltaic battery. The mixed saliva of the mouth, with the decomposing particles of food which it contains, together with the acid of fruits, drinks, etc., will answer to the fluid of the cell. So, of course, the greater the departure of the oral fluids from a natural and purely neutral, or slightly alkaline saliva, the greater the chemical effect on the dentos.

Voltaic electricity having the power to disintegrate even metals, as well as animal and vegetable substances, it also has the power to disintegrate dentos.

The rapidity with which this may be effected must depend on the quantity and intensity of the battery. Ordinarily, the healthy mouth is *not* in a condition to decompose dentos, *appreciably*. Where the teeth are all sound, and the oral fluids are in a perfectly physiological condition, the mouth is not a voltaic battery, for everything being in harmony no chemical action takes place. But thermo-electrical currents may be produced by sudden changes of temperature. When hot or cold drinks or food is received into the mouth, the temperature is not changed in all portions at the same time, and that which takes place between metals on heating them, may also take place here, and cause electrical currents. There are more or less hiding places in the fissures of the teeth, and between the latter, in which the mixed saliva has sufficient power to set up electrical action. The result of my experiments show that even river water, by its action on metals, will produce a decided current, which can be seen in the deflection of the needle in a galvanometer.

There is, probably, more or less electricity evolved in the ordinary mouth nearly all the time, more especially if there is a tooth plugged with *any metal whatever*.

Electrical currents probably do no harm, excepting where by their quantity, intensity, and constant direction they produce chemical disintegration of dentos, or produce pathological changes in the pulp, or contents of the dentine tubes.

When plugs leak, so as to allow the mixed saliva to permeate between the walls of the cavity and the plug itself, we have in each such tooth a battery; and there are as many batteries, of a single cup each, in the mouth, as there are such leaky plugs.

We have this same condition of things when a plate of impure zinc is employed in a voltaic battery. Between the particles of iron which the zinc may contain, and the zinc itself, innumerable batteries are formed; the sulphuric acid acting upon these metals with different degrees of energy.

Although voltaic batteries are usually made with one metal which is not acted upon at all by an acid, and another metal which is very easily acted upon, yet we will find that currents of electricity can be evolved by the use of two dissimilar metals, *both* of which are acted upon by chemical agents, or between them and other substances.

The electrical current passes from the positive element to the negative one; and that element is positive which is the *most* easily acted upon by the fluid in which the elements are immersed; and that element is negative which is less acted upon than the positive one with which it is in circuit.

Changing the fluid of the cup will often change the relative positions of the elements. Lead is *positive* to copper in nitric acid; but in a solution of sulphide of sodium *copper* is positive to lead.

In voltaic electricity those substances which are least easily acted upon, chemically, are called the least potentials—that is, having the least electro positive qualities. The elements of batteries which are sometimes found in the mouth, are enamel, dentine, muscle, gold, dental amalgam, tin, gutta percha, oxy-chlo. zinc. As will be seen in the experiments, accompanying this paper, the relation which these substances hold to each other, may vary in potential, according to the fluid in which they are immersed. Any of the above substances touching each other in the mouth, form batteries of one or many cells, the intensity or power of which are determined by the condition of the oral fluids, and the relative positions which the substances sustain to each other. Each metallic plug forms a battery by its union with the tooth in which it is placed,

If the plugs are water-tight then the electrical action is limited to the margins of the plug; but if the plug is leaky then there is electrical action set up within the cavity at every point at which the filling and the dentos touch.

Dentos is a very bad conductor of electricity, and therefore in my experiments with the galvanometer the record of deflection of the needle does not indicate the power of dentos as a positive element in the oral battery.

In regard to the facility with which the following substances dissolve or lose in weight, in acid solutions, the following order is obtained by my experiments, viz: Dentos, or Tooth Substance; Smith's Oxy. Cl. Zinc; Fletcher's Enamel; Hill's Gutta Percha; Tin; Dental Amalgam; Gold.

If the first four were better conductors they would undoubtedly show a deflection of the galvanometer needle, when associated with gold in a battery, much greater than the last two. But if the list is made up from battery and galvanometer experiments, I have to place them according to the following order as to their potential on the positive scale: Tin, Amalgam, Dentos, Muscle, Ox. Cl. Zinc, Hill's Gutta Percha, Gold.

But this would evidently be incorrect, because the electrical action between two substances is none the less on account of the *resistance* offered to the passage of the current.

Zinc and Platinum make a powerful battery, but if the current has to pass through a poor conductor, such as dentos, or Hill's gutta percha, the deflection of the needle would show a very feeble current passing. In this case the electric current is changed into heat.

When two elements are acted upon chemically, but in different degrees, the one which is positive in relation to the other, when placed in the circuit, will be dissolved at the expense, so to speak, of the negative element. Example: Place a copper plate and a zinc plate in dilute sulphuric acid, joining them by a wire, or allowing the metals to touch each other at the upper end. The copper will be negative to the zinc, consequently the copper will not be chemically acted upon, and will lose none of its weight; but the zinc will be acted upon, lose weight, and send a current from itself to the copper; the action is on the zinc alone. In a stronger fluid both might lose weight.

Every material which is used for plugging teeth is negative to dentos; consequently, dentos is placed in a critical position by every plug which it receives, because it is *positive* to every dental plug. The only safety for a plugged tooth is in a perfectly neutral condition of the saliva. That material which stands nearest to dentos on the potential scale would give the greatest assurance of safety, so far as disintegration of tooth substance could be caused by electrical currents, and by its association with a negative substance to form a battery.

The most powerful current doubtless would be within a plugged cavity which contained a plug admitting the mixed saliva of the mouth. The latter would soon become acidulous, and setting up action upon the dentos, there would be currents at every point of contact of dentos and plug.

The current between a plug and dentos is so feeble that it is not usually noticed by the person in whose mouth the action is taking place.

When there are plugs of different substances in teeth, there is no appreciable current developed between the plugs, unless they are in contact. Even if in the same tooth, the dentos is too poor a conductor to convey an electric current with any degree of intensity.

When two metallic plugs touch each other, either in the same tooth, or in different teeth, a current is evolved which is constant, however feeble.

This current may be constantly interfered with and the current more or less stolen by the contact of the tongue or cheek, or contact of food: for these are conductors of electricity, and very good ones, too.

In my experiments, I found muscle a very good conductor, and also a very good element for the production of a current.

A piece of muscle impacted between two teeth, or crowded into the crevice of a tooth, will produce considerable of a current. When there are two plugs facing each other, of different metals on the proximal surfaces of teeth, and a particle of meat is impacted between them, a current of electricity is evolved which is usually so annoying that the removal of the substance seems an immediate necessity to the individual.

This is more especially the case if one of the plugs approaches the dental pulp closely.

It certainly is a very comfortable provision of nature that dentos *is* such a poor conductor of electricity.

Between a gold plug and dentos there would be a stronger current produced than between amalgam and dentos, or tin and dentos. The current would be from the dentos through the liquid to the plug in the case of either of these metallic substances. But it would be strongest between gold and dentos, because the latter is further from gold on the potential scale than either tin or amalgam.

In some way, not yet understood, the voltaic current alone, by itself, promotes disintegration. Not only that, but it *produces* disintegration. Example: When the ends of two conductors from a battery cell are immersed in another vessel containing water, the latter is decomposed, and its hydrogen and oxygen may be caught, measured and tested.

By this method various solutions are decomposed, and the process is called *Electrolysis*—the fluid undergoing decomposition being called an electrolyte.

A healthy tooth being situated in a corrosive oral fluid, is like a battery of one cup, where there is *one* metal and two fluids. And such is a "*Daniel's Cup.*" There is a mild, healthy plasma in the substance of the plasma in the dentos, and a *corrosive* liquid outside; the two liquids *touch*, but do not commingle, and so there is electricity evolved, and the current is from the *outside* of the tooth to its interior.

May not this fact give us a hint in regard to the inception and progress of dental decay?

This oral battery would be still more powerful if the dentos was chemically eroded or necrosed at some point. We often find the enamel porous and easily cut at some point of proximate contact, before there is a cavity at all. Now when this porous dentos becomes filled with the corrosive fluid formed from food, mucus, saliva and acids, and it enters the tubuli so far as to touch the blood plasma, a condition for active and powerful electric action is produced.

The current produces decomposition, and so the corrosive fluid is kept constant.

In this way may it not be that decomposition of the tube plasma, beyond the territory of actual decay, causes subacute inflammation of the pulp, which may lead either to the death of that organ, or stimulate the latter to the production of new dentos to protect itself from the advancing decay. And may not this electric current cause a decomposition of lime salts from the blood plasma in the dentine tubes? What we know of Electrolysis suggests the thought.

Dentos being such a poor conductor of electricity, the current could only be feebly passed from tooth to tooth, where they were all in actual contact, even when the teeth are plugged with metals far distant from each other on the potential scale, namely, between gold and tin.

In the buccal surfaces, plugs which might form a strong battery, as gold and tin, the mucous membrane of the cheek would form a better current than the teeth themselves, but then such a conductor would practically carry the current all over the mouth. and being so diffused would not be felt, and would also lose its power to do harm.

When a plug oxidises, voltaic action diminishes or ceases. Between gold and tin, or between gold and amalgam, the current is diminished just in accordance with the amount of oxide remaining on the plug; for the latter becomes less positive to the gold, and less negative to the dentos.

The changing thermal conditions of the mouth may perhaps change the relative potential of plugs if they are near to each other on the scale.

Gold, which is so far removed from dentos, tin, amalgam, etc., would probably maintain its negative position in the oral battery. But tin, dentos, etc., might perhaps change places under some thermal conditions.

Electrical currents are produced by thermal changes. Then may not pathological effects be produced in *undecayed* teeth by frequent extreme changes, so as to result in exostosis, calcification of the pulp, or necrosis of the latter.

*Example.*—A piece of antimony and a piece of bismuth, with one end of each placed in contact, and heated, even with the warmth of the tongue, will develop a current which will deflect the magnetic needle. Extremes of temperature favor the development of the current. If one metal be heated, while the other is cooled by ice, a more powerful current is evolved than otherwise.

Two metals of different temperatures, immersed together in a liquid, will develop a weak current of electricity.

Two plates of the same metal, at different temperatures, immersed in one liquid, develop a weak current of electricity which passes from the hot to the cold metal.

A cold cathode immersed in a warm depositing liquid causes a deposit to take place with more facility than when immersed at the same temperature.

Usually no current will be evolved between metals unless there is contact. But "Guthrie" says a current can be produced by two metals and two liquids *without* contact of dissimilar metals. He also says that contact between a single metal and a liquid can produce electrical excitement.

Texture of metallic surface affects polarity. A rough surface and a smooth surface with the *same* metal, will cause a current to pass from the rough to the smooth one. The rough surface is positive to the smooth one. Tin and gold are sometimes used in making cylinders. In such cases the tin should always be on the *outside* of the gold, so that the tin may come next to the dentos. For although a current would pass from the dentos to the tin, yet it would not be nearly as strong, as though the gold was next the dentos. In packing such cylinders the two metals would become mixed on the surface of the plug, and then the latter would partake of the nature of an alloy. This alloyed surface would be less negative than gold. It would be better than gold for *preservative* purposes and better than tin for masticatory purposes. Thus the usefulness of this method would be limited to *grinding* surfaces.

But a better method to use tin and gold in the same cavity is to employ cylinders of tin only, and of gold only; always keeping the tin next to the walls of the cavity alone, and using only gold in the center of the plug.

The galvanometer experiments which accompany this article can be regarded as only approximately true so far as locating on the potential scale the different substances, other than *metals*, which occupy the mouth.

The proper mode in which to obtain the knowledge of their potential position is to take equal superficial surfaces of all of them, weigh them after having soaked in water until they refuse to gain more in weight, and then put them into various chemical corrosive fluids, under the same conditions; the loss in weight, sustained by each substance, will enable us to place it in its true position on the potential scale. By trying a great number of experiments, I believe I have arrived at the truth. That you may judge for yourselves I herewith append a copy of these experiments.

If I have stated nothing but that which is regarded as true by experts in electrical science; and if my experiments are confirmed by others, then, wherever these truths become widely known, there must result a great modification in modes of practice.

Gold can then no longer hold its present position as an arrester of dental decay, it must share its honors with amalgam and tin, and, in some cases, take a position inferior to both. As gold, united to dentos, forms a more powerful battery than does dentos united with any other plugging material used by our profession, it is only in the most healthy and cleanly of mouths that it is admissable. The hygienic habits of the patient must be also considered, for if acids are freely used, and hot and cold drinks alternately in quick succession, then also gold is the worst plug that can be employed in the teeth.

I am speaking *only* of the *preservation* of the teeth, and not of dental æsthetics.

As for myself, I hope to use the knowledge which I have gained on this subject, for the great good of my patients, without regard to my *former* opinions, or those of others.

In my experiments I used a battery of a single cell and a galvanometer, taking care to eliminate every source of error.

In speaking of the elements, the positive one will always be written first.

GALVANOMETER AND BATTERY.

*Nitric Acid* 1 *part, Water* 20=$\frac{1}{20}$.

Tin and gold—deflection of the galvanometer..........90°

Amalgam and gold..................................82°

Muscle and gold...................................20°

Tooth and gold....................................20°

Tooth and tin.....................................60°

Tooth and amalgam.................................10°

Tooth and muscle..................................[illegible]5°

*Hydro Chloric Acid*, $\frac{1}{20}$.

Tin and gold. deflection of needle................73°

Amalgam and gold..................................70°

Muscle and gold................................... 5°

Tooth and gold....................................22°

Tooth and tin.....................................60°

Tooth and amalgam.................................10°

*Sulphuric Acid*, $\frac{1}{20}$.

Tin and gold, deflection of needle................77°

Amalgam and gold..................................80°

Muscle and gold................................... 5°

Tooth and gold....................................10°

Tooth and amalgam................................. 5°

Tooth and tin.....................................15°

Tooth and muscle.................................. 2°

*Strong Cider Vinegar.*

| | |
|---|---|
| Tin and gold, deflection of needle | 40° |
| Amalgam and gold | 25° |
| Muscle and gold | 8° |
| Tooth and gold | 10° |
| Tin and muscle | 5° |
| Amalgam and muscle | 2° |

*Water of Ammonia.*

| | |
|---|---|
| Tin and gold, deflection of needle | 15° |
| Amalgam and gold | 3° |
| Tooth and gold | 0 |
| Tooth and tin | 0 |
| Tooth and amalgam | 0 |

*Fresh Saliva.*

| | |
|---|---|
| Tin and gold, deflection of needle | 3° |
| Amalgam and gold | 12° |

*Saliva and Tobacco Juice.*

| | |
|---|---|
| Tin and gold, deflection of needle | 2° |
| Amalgam and gold | 9° |
| Muscle and gold | 3° |
| Tin and muscle | 8° |
| Amalgam and muscle | 7° |

I have already remarked, that substances standing very near to each other on the potential scale are liable to exchange places with each other on the list, by being placed in a different liquid.

I will call your attention to this fact in regard to tin and Fletcher's alloy, the latter being the one which I used in galvanometer experiments. This alloy, or amalgam, stands between gold and tin; that is, it gives the least deflection of the needle when united with gold in a battery having for a fluid either dilute nit. acid; hy. cl. acid; water of ammonia, or cider vinegar. But tin stands between Fletcher's alloy and gold, when united to gold in saliva and tobacco juice, pure fresh saliva, or sulph. acid.

*Immersions of substances in acids for specified times to ascertain per centage of loss.*

Ivory, dentine (dense) $7\frac{1}{2}$ pr. ct. loss in Cider Vinegar, 24 h'rs.

" " " 8 " " Nit. Acid, $\frac{1}{20}$, 24 "

Enamel of tooth 40 per cent. loss in Nitric Acid, $\frac{1}{20}$, 24 "

" " 49 " " " " $\frac{1}{20}$, 24 "

Smith's Ox. Cl. Zinc, 35 pr. ct. loss in Nit. Acid, $\frac{1}{20}$, 24 "

" " 27 " " Cider Vin., 24 "

" " 41 " " Sulp. Acid, $\frac{1}{20}$, 24 "

" " 21 " " Hy. Cl. " $\frac{1}{20}$, 24 "

Fletcher's Enamel. (oz. cl. zinc) 8 pr. ct. loss in Vinegar, 24 "

" " 19 per cent. loss in Nit Acid, $\frac{1}{20}$, 24 "

Hill's Gutta Percha, $\frac{1}{2}$ per cent. loss in Nit. Acid, $\frac{1}{20}$, 24 "

Johnston's " oo " " " $\frac{1}{20}$, 24 "

" " $1\frac{1}{8}$ " " Hy. Cl. Acid, $\frac{1}{20}$, 24 "

Tin, lost oo in Vinegar,..........................................7 d'ys.

" " $\frac{1}{8}$ per cent, in Lemon Juice..........................7 "

" " $\frac{2}{3}$ " Hy. Acid, $\frac{1}{20}$..........................24 h'rs.

" " 7 " Nit. Acid, $\frac{1}{20}$..........................24 "

" " $\frac{1}{2}$ " Sul. Acid, $\frac{1}{20}$..........................24 "

Amalgam lost $\frac{1}{7}$ of one per cent in Lemon Juice........24 "

" " oo in Sulp. Acid, $\frac{1}{20}$..........................24 "

" " $\frac{1}{15}$ per cent. in Hy. Acid, $\frac{1}{20}$................24 "

" " $1\frac{1}{2}$ per cent. Nit. Acid, $\frac{1}{20}$....................14 "

Fletcher's Amalgam, 400 c.g., lost oo in Hy. Cl. Ac., $\frac{1}{10}$, 50 d'ys.

Amalgam, 1500 c. g., lost $\frac{1}{20}$ per cent. in Sulp. Ac., $\frac{1}{20}$, 14 "

" " " " $\frac{1}{33}$ " Hy. Cl. Ac., $\frac{1}{20}$, 14 "

285 c. g., Mercury, lost in 24 hours oo in Lemon Juice.

1 c. g. in Cider Vinegar,

5 c. g. in Sulphuric Acid, $\frac{1}{20}$

6 c. g. in Hy. Cl. Acid, $\frac{1}{20}$

11 c. g. in Nitric Acid, $\frac{1}{20}$

The above are only representative experiments. The whole number is too tedious to report, amounting as they do to several hundred.—*Missouri Dental Journal.*

## Figures and Tables

**Figure f1:**